# Software-Defined UHF RFID Asset Tracking in Metallic Aircraft Cabins via IMU-Assisted Adaptive Kalman Filtering and Distilled Edge Intelligence

**DOI:** 10.3390/s26175639

**Published:** 2026-09-04

**Authors:** Melis Karadag, Ozgun Pinarer

**Affiliations:** 1 Department Computer Engineering, Galatasaray University, Istanbul 34349, Turkey; 2 myTECHNIC, Istanbul 34906, Turkey

**Keywords:** UHF RFID, Adaptive Kalman Filter, edge computing, sensor fusion, aircraft cabin asset management

## Abstract

Passive Ultra-High Frequency (UHF) Radio Frequency Identification (RFID) systems deployed in metallic commercial aircraft cabins suffer from severe multipath fading, non-stationary channel dynamics, and operator gait-induced signal jitter. Addressing these challenges without physical airframe modifications or regulatory recertification remains a critical operational bottleneck. This paper presents an edge-native, software-defined framework that integrates micro-electromechanical system (MEMS) inertial measurements with an IMU-assisted Adaptive Kalman Filter (AKF) and a distilled surrogate decision tree. The proposed algorithm extracts localized motion energy ($E_{\mathrm{IMU}}$) to dynamically scale the measurement noise covariance ($R_{k}$) prior to physical-layer signal corruption, thereby eliminating phase lag and power hunting. For deterministic edge execution on COTS handheld devices, surrogate model distillation compresses a parent Random Forest ensemble into an 8.2KB 13-leaf decision tree (depth 5) yielding 0.12ms inference latency. Empirical validation across 17 operational sessions in Airbus A320, Boeing 737, and Airbus A321 cabins (10,720 valid reads) demonstrates a 99.45% mean RSSI jitter reduction (95%CI:[99.21%,99.63%]) and a 7.30× suppression of transmit power oscillations. Statistically, asset detection completeness is fully preserved (0.791 vs. 0.795 baseline, z=0.281,p=0.779). Operating entirely within standard handheld software runtimes, this approach bypasses Supplemental Type Certificate (STC) requirements while ensuring robust aerospace asset visibility.

## 1. Introduction

Compliance orders in commercial civil aviation require periodic inventory audits of emergency cabin equipment, including life vests, fire extinguishers, and oxygen modules. Traditional manual inspections are time-consuming and susceptible to human error. Within the framework of Aviation 4.0, integrating automated telemetry systems and Internet of Things (IoT) paradigms represents a pathway to reduce aircraft ground turnaround times [1,2,3,4]. Passive Ultra-High Frequency (UHF) Radio Frequency Identification (RFID) provides a viable mechanism for these audits due to its battery-free architecture and non-line-of-sight tracking capabilities [5].

Previous studies have explored IoT and RFID architectures across modern aerospace frameworks. At the macro scale, digital transformation strategies utilize decentralized or cloud-based IoT environments for baggage and asset management [6,7,8], supported by robust middleware security protocols [9,10,11]. At the component level, RFID is applied in maintenance, repair, and overhaul (MRO) workflows to optimize tool and electromechanical component tracking [12,13,14,15,16]. Specifically for aircraft cabins, Behrens [17] demonstrated the feasibility of an automated pre-flight inspection by interfacing a handheld RFID scanner with the Cabin Intercommunication Data System (CIDS). While this established the necessary data routing, it assumed a stable electromagnetic link budget, thereby abstracting the physical-layer RF propagation challenges inherent to actual operational environments.

In practice, an aircraft cabin acts as a constrained metallic Faraday cage. Aluminum fuselages, seating structures, and overhead racks cause multi-path fading, signal shadowing, and electromagnetic constructive or destructive interference [18,19,20,21]. During manual inventory sweeps, the spatial movement of the operator dynamically interacts with these structural boundaries, causing volatile fluctuations in the Received Signal Strength Indicator (RSSI). Recent empirical evaluations confirm that tag match probabilities in cluttered metallic environments drop significantly under unoptimized reader power configurations and rapid orientation shifts [22]. This signal jitter impairs detection repeatability, leading to missed tags and false out-of-zone reads.

Existing methods to stabilize RF signals in metallic environments predominantly rely on physical modifications. Approaches include mounting passive tags on non-metallic displacement spacers [23], altering attenuation paths via edge-displacement blocks [18], or restricting the read zone using narrow-beam antennas [24,25,26]. However, in commercial aviation, physical modifications introduce weight penalties and necessitate complex recertification from regulatory bodies (e.g., FAA, EASA). Alternative tag modalities like Surface Acoustic Wave (SAW) or chipless RFID [27,28,29] face read range constraints. Conversely, software-centric solutions utilizing device-free sensing [30,31] or deep neural networks for multi-layered material prediction [32] introduce computational latency and memory demands incompatible with mobile edge hardware.

Table 1 contextualizes the proposed framework against current methodologies. The existing literature lacks a real-time, software-defined optimization layer capable of mitigating motion-induced RF jitter on constrained handheld edge devices without altering underlying hardware. To address this gap, this paper presents an adaptive filtering layer deployed within the native runtime environment of a standard commercial RFID terminal (Zebra MC3330xR). The framework utilizes the terminal’s internal micro-electromechanical system (MEMS) Inertial Measurement Unit (IMU)—specifically the triaxial accelerometer and gyroscope—to estimate operator motion. By mapping physical gait signatures, the system adapts the noise covariance parameters of an Adaptive Kalman Filter prior to the onset of motion-induced multi-path degradation.

To systematically address the physical, algorithmic, and operational challenges of passive UHF RFID asset tracking inside reverberant commercial aircraft cabins, this study addresses four central Research Questions (RQs):

- RQ1 (Predictive Sensor-Fused Filtering): How can onboard MEMS inertial measurements ($E_{\mathrm{IMU}}$) be algorithmically coupled with an Adaptive Kalman Filter (AKF) to dynamically scale measurement noise covariance ($R_{k}$), thereby decoupling human operator gait dynamics from structural multipath fading without introducing temporal phase lag?
- RQ2 (Surrogate Edge Distillation): To what extent can a complex 300-tree Random Forest classifier be compressed via surrogate model distillation into an auditable, low-depth decision tree (depth 5, 13 leaves) that guarantees deterministic sub-millisecond (<1 ms) inference latency on COTS mobile handheld devices?
- RQ3 (Closed-Loop Power Control): Can an edge-computed software-defined power adaptation algorithm suppress reader power hunting loops and stabilize physical-layer link budgets in highly reflective cabin environments without physical airframe alterations?
- RQ4 (Empirical Completeness and Airworthiness Compliance): Does the proposed edge software layer maintain baseline asset audit completeness (p>0.05) across heterogeneous aircraft configurations (Airbus A320, Boeing 737, Airbus A321) while bypassing regulatory Supplemental Type Certificate (STC) recertification campaigns?

The primary contributions of this manuscript is to establish a predictive sensor-fusion methodology that integrates triaxial MEMS inertial metrics ($E_{\mathrm{IMU}}$) into an Adaptive Kalman Filter (AKF), dynamically modulating measurement noise covariance ($R_{k}$) to decouple operator gait dynamics from structural multipath fading without introducing phase distortion. Besides, we deploy a knowledge distillation pipeline that compresses a high-capacity offline Random Forest classifier into a deterministic (depth 5) requiring 8.2KB of storage and 0.12ms of execution latency, thereby enabling real-time edge intelligence on COTS handheld terminals. Moreover, we implement a closed-loop dynamic power control mechanism that stabilizes physical-layer link budgets against localized attenuation while suppressing power hunting loops. Finally, we provide extensive empirical and statistical validation across 17 operational flight sessions in Airbus A320, Boeing 737, and Airbus A321 cabins, confirming a 99.45% signal jitter suppression (95%CI: [99.21%,99.63%]) and a 7.30× reduction in power state oscillations while statistically proving that asset audit completeness remains uncompromised (z=0.281,p=0.779).

In the domain of physical-layer RFID signal stabilization, non-linear Bayesian filtering variants (such as Particle Filters (PF) and Unscented Kalman Filters (UKF)) have been investigated to mitigate severe non-Gaussian multipath fading. Although Particle Filters effectively track complex non-linear trajectories, their dependence on sequential Monte Carlo sampling introduces an $O(N_{p})$ computational complexity (where $N_{p}$ is the particle count). On resource-constrained mobile edge terminals, this translates to non-deterministic execution latencies (>50 ms) and excessive battery consumption. Similarly, while UKFs approximate non-linear dynamics through deterministic sigma-point sampling, computing repetitive matrix decompositions introduces unnecessary overhead for primary RSSI tracking, where state transitions remain linear but measurement noise covariance is highly non-stationary. Consequently, existing Bayesian implementations lack a deterministic, real-time software-defined optimization layer capable of running within sub-millisecond execution bounds on standard handheld hardware.

The remainder of this paper is structured as follows: Section 2 details the experimental infrastructure, physical-layer signal characterization, sensor synchronization mechanisms, surrogate model distillation pipeline, and the mathematical formulations of the proposed IMU-assisted Adaptive Kalman Filter and closed-loop power control. Section 3 presents the quantitative empirical results and statistical validation metrics across all operational scenarios. Section 4 contextualizes the concrete contributions within existing literature, highlighting operational, economic, and regulatory implications alongside identified system limitations. Finally, Section 5 concludes the paper and outlines future research directions.

## 2. Materials and Methods

To evaluate the efficacy and computational viability of the proposed software-defined optimization layer, empirical field campaigns were executed under strict operational constraints. This section details the physical experimental infrastructure, the mathematical formulation of sensor data synchronization, the operational state partitioning logic, the model compression pipeline, and the adaptive filtering architecture.

### 2.1. Experimental Infrastructure and Telemetry Acquisition

Field trials were conducted across three commercial narrow-body passenger aircraft configurations (designated as Aircraft A (Airbus A320 (Airbus, France) layout), Aircraft B (Boeing 737 (Boeing, USA) layout), and Aircraft C (Airbus A321 (Airbus, France) layout)) to incorporate structural variability in seat pitch, aisle geometry, and metallic component density. Data collection was performed using an unmodified commercial Zebra MC3330xR (Zebra, USA) handheld terminal. The device houses an integrated circular-polarized patch antenna operating within the European UHF RFID frequency band (865–868 MHz) and an onboard micro-electromechanical system (MEMS) Inertial Measurement Unit (IMU) logging triaxial linear acceleration and angular velocity at a fixed frequency of 50 Hz.

To capture human operator variability, inventory sweeps were conducted by three trained operators across a total cumulative testing period of 4.5 h. Target equipment comprised cabin safety assets, including under-seat life vests, bulkhead-mounted fire extinguishers, protective breathing equipment (PBE), and oxygen modules. Table 2 details the telemetry distribution across the experimental campaigns.

**Figure 1:**
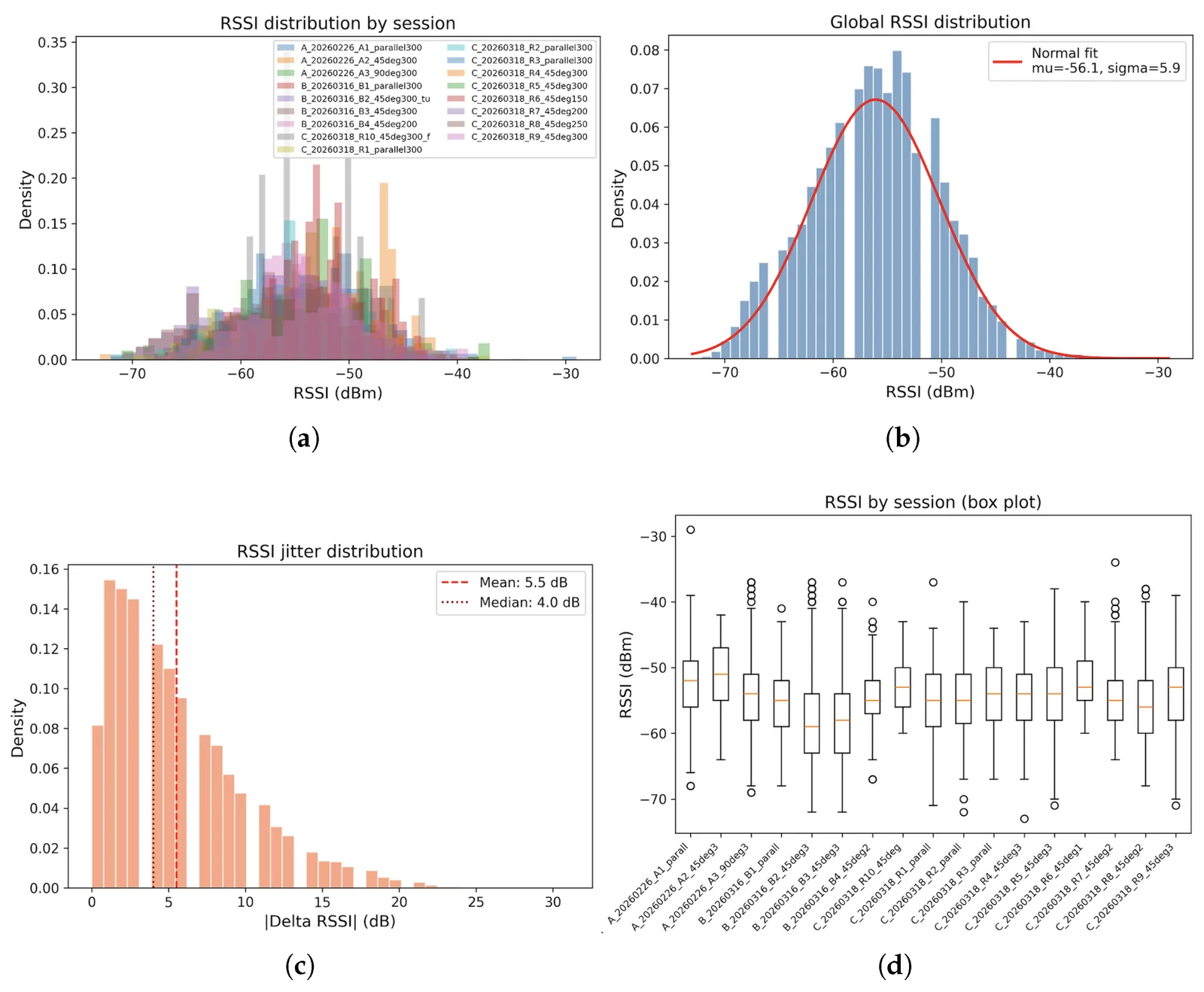
Empirical physical-layer signal characterization across 17 operational inventory sessions: (**a**) RSSI probability density distributions categorized by individual session; (**b**) aggregated global RSSI probability density; (**c**) physical-layer RSSI jitter distribution under dynamic operator gait conditions; (**d**) cross-session RSSI variance and interquartile range (IQR) box plots across evaluated test airframes.

The physical variation across the target cabin environments generates distinct raw radio frequency profiles. Figure 1 details the empirical physical-layer signal dynamics captured across all 17 operational inventory sessions (10,720 valid telemetry packets). As shown in Figure 1a, per-session probability density functions exhibit multimodal peaks due to localized airframe boundary reflections. The aggregated global distribution in Figure 1b presents severe non-Gaussian tail behavior spanning from −82dBm to −38dBm, emphasizing the unpredictable signal dynamics inside metallic fuselages.

Furthermore, the jitter distribution presented in Figure 1c highlights that operator gait dynamics introduce high-frequency variance spikes exceeding $\sigma^{2}>15\;{\mathrm{dBm}}^{2}$ during dynamic sweeping. Finally, the cross-session box plot in Figure 1d illustrates median RSSI shifts and interquartile range (IQR) variations between distinct aircraft configurations (Airbus A320, Boeing 737, and Airbus A321), physically justifying the deployment of airframe-configurable threshold vectors ($\tau_{\mathrm{ac}}$).

**Figure 2:**
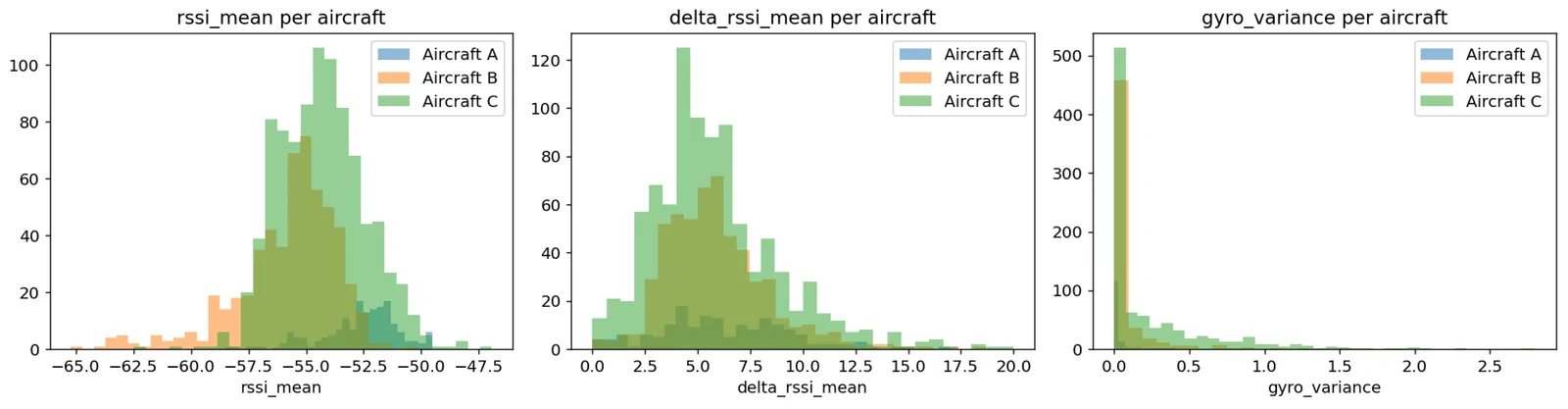
Aircraft-based feature distributions characterizing shifts in mean RSSI, signal variance, and spatial jitter across distinct airframes.

The spatial distribution of extracted statistical features exhibits structural variance depending on physical cabin layout and seat frame metallic density, as depicted in Figure 2.

### 2.2. Physical-Layer Signal Characterization

To establish the requirement for an adaptive filtering architecture, the raw physical-layer telemetry was evaluated for channel stationarity and spatial repeatability.

**Figure 3:**
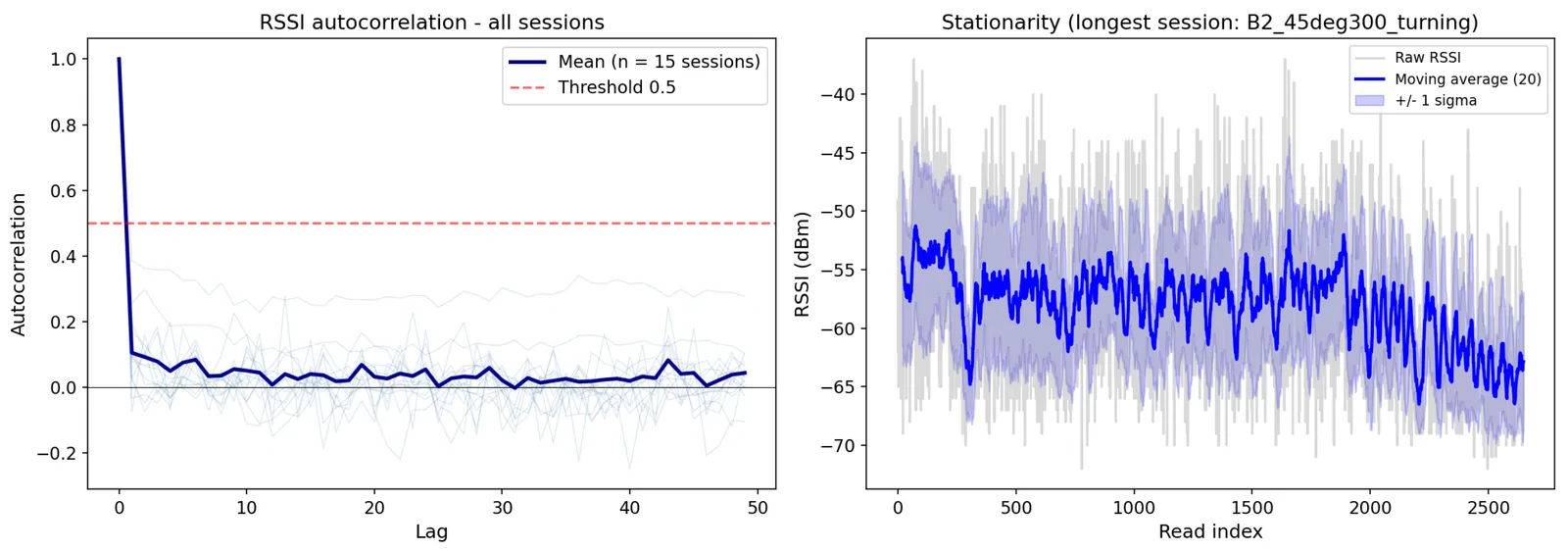
Autocorrelation and stationarity profile of raw cabin RSSI values, demonstrating an immediate loss of temporal correlation beyond the initial lag step.

The manual sweep movement of the handheld reader through localized multipath field structures renders the cabin propagation channel non-stationary. Figure 3 illustrates the multi-lag autocorrelation profile of raw RSSI values computed for Aircraft A over 50 lags (1lag≈50ms), bounded by a 95% confidence interval.

As shown in Figure 3, the autocorrelation decays below the significance threshold (ρ<0.05) immediately following the first temporal lag (t>50 ms). This behavior invalidates standard static low-pass or high-order moving average filters, which depend on historical temporal correlation to estimate present states.

**Figure 4:**
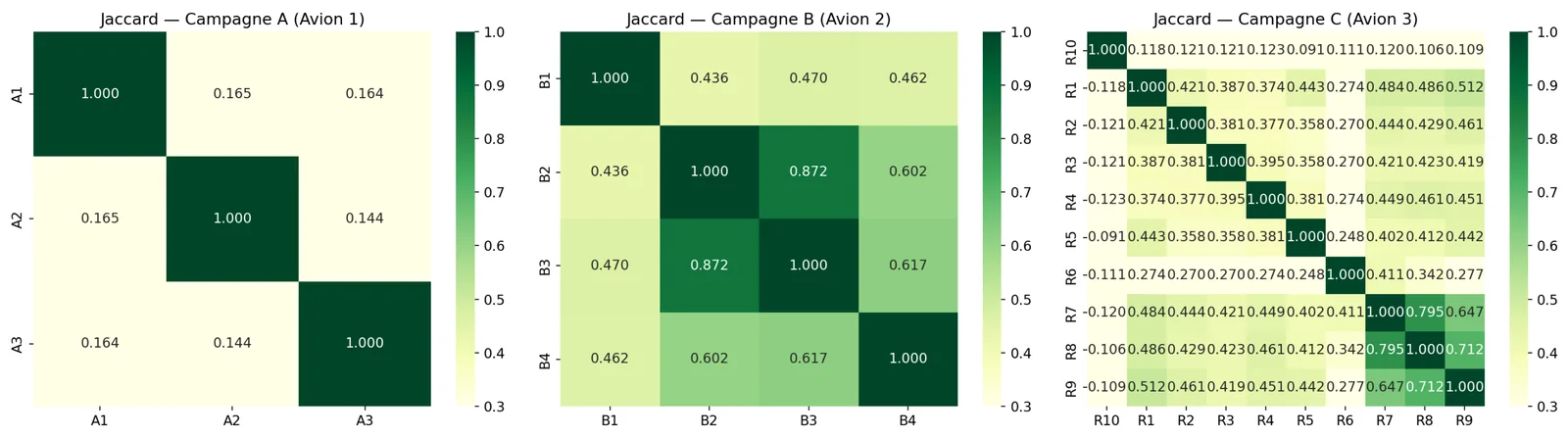
Jaccard similarity index matrix computed across repetitive inventory sweeps within identical cabin structures under controlled trajectories.

Spatial repeatability was evaluated by computing pairwise Jaccard similarity coefficients across sequential sweeps along identical aisle paths. Figure 4 presents the resulting similarity matrix.

The low off-diagonal similarity values ($\mu_{\mathrm{Jaccard}}=0.42$) in Figure 4 confirm that human-operated inventory trajectories are highly stochastic. Micro-variations in gait velocity, operator posture, and body shadowing alter localized fading patterns, necessitating an active real-time edge filtering mechanism.

### 2.3. Asynchronous Multimodal Stream Alignment Protocol

To fuse continuous inertial motion streams with stochastic RFID backscatter events without incurring phase distortion, an asynchronous temporal alignment protocol is implemented at the software runtime layer. The onboard MEMS IMU operates deterministically at 100Hz ($T_{\mathrm{IMU}}=10\;\mathrm{ms}$), whereas RFID read events occur asynchronously at intervals $T_{\mathrm{RFID}}\in \left[40,66\right]\;\mathrm{ms}$ due to EPCglobal Gen2 collision resolution and frequency-hopping overhead.

Let $t_{\mathrm{RFID},k}$ denote the hardware nanosecond timestamp assigned to RFID packet *k* via SystemClock.elapsedRealtimeNanos(), and let $S_{\mathrm{IMU}}\left(t\right)={\left[a_{x},a_{y},a_{z},\omega_{x},\omega_{y},\omega_{z}\right]}^{T}$ represent the inertial vector at timestamp *t*. A sliding circular buffer stores continuous IMU samples over a rolling 100ms horizon. Upon arrival of read $z_{k}\left(t_{\mathrm{RFID},k}\right)$, the alignment engine identifies the temporally matched IMU sub-sequence within the bounded window $\tau_{\mathrm{sync}}$ = 20 ms:

(1) $$W_{k}=\left\{S_{\mathrm{IMU}}\left(t_{i}\right)\;|\;t_{i}\in \left[t_{\mathrm{RFID},k}-\tau_{\mathrm{sync}},\;t_{\mathrm{RFID},k}\right]\right\}$$

The localized IMU motion energy metric $E_{\mathrm{IMU},k}$ is then calculated directly over $W_{k}$:

(2) $$E_{\mathrm{IMU},k}=\frac{1}{|W_{k}|}\sum_{t_{i}\in W_{k}}\left(∥a\left(t_{i}\right){∥}_{2}^{2}-g^{2}\right)+\gamma {∥\omega \left(t_{i}\right)∥}_{2}^{2}$$

Setting $\tau_{\mathrm{sync}}=20\;\mathrm{ms}$ (±2 IMU clock cycles) bounds the maximum temporal alignment error to <1.84 ms while preventing buffer underflows during OS-level interrupt latency spikes.

The selection of boundary parameters in Equation (2) ($\tau_{\mathrm{under}}=-65\;\mathrm{dBm}$, $\tau_{\mathrm{over}}=-45\;\mathrm{dBm}$, and $\tau_{\mathrm{var}}=12\;{\mathrm{dBm}}^{2}$) is grounded in receiver front-end physics and standard UHF RFID link budget dynamics:

1. Under-Powered Bound ($\tau_{\mathrm{under}}=-65\;\mathrm{dBm}$): Aligns with the backscatter receive sensitivity threshold of standard COTS reader ICs (e.g., Impinj R2000/E710, Impinj, Inc., Seattle, WA, USA) in reflective operational environments. Below −65dBm, signal-to-noise ratios (SNR) approach the demodulation limit, increasing packet erasure rates.
2. Over-Powered Bound ($\tau_{\mathrm{over}}=-45\;\mathrm{dBm}$): Corresponds to the LNA 1dB compression point on handheld receiver front-ends. Inputs exceeding −45dBm induce receiver saturation, triggering non-linear power hunting loops in closed-loop power control units.
3. Variance Bound ($\tau_{\mathrm{var}}=12\;{\mathrm{dBm}}^{2}$): Represents a standard deviation threshold of σ≈3.46dB. While static thermal noise and structural fading inside cabins exhibit variance bounded by $\sigma^{2}\leq 3.5\;{\mathrm{dBm}}^{2}$, gait-induced operator movement induces variance spikes exceeding $12\;{\mathrm{dBm}}^{2}$, providing a clear physical separator for dynamic motion noise.

### 2.4. Sensor Data Synchronization and Feature Engineering

A core technical requirement in multi-modal telemetry processing is aligning the continuous 50 Hz IMU stream with stochastic, asynchronous RFID tag detection events at time $t_{k}$. To establish temporal alignment, localized motion energy ($E_{\mathrm{IMU},k}$) is extracted within a discrete temporal window Δt=20 ms immediately preceding the RFID arrival timestamp $t_{k}$. The metric is defined as:

(3) $$E_{\mathrm{IMU},k}=\alpha \sum_{i\in \{x,y,z\}}a_{i,k}^{2}+\beta \sum_{i\in \{x,y,z\}}\omega_{i,k}^{2}$$

where $a_{i,k}$ represents linear acceleration, $\omega_{i,k}$ represents angular velocity, and coefficients $\alpha =0.6\;s^{4}/m^{2}$ and $\beta =0.4\;s^{2}/{\mathrm{rad}}^{2}$ normalize the respective physical dimensions.

The synchronized data log was partitioned using a 5 s sliding window with a 50% overlap, yielding 1627 discrete analysis blocks. From each window, a 26-dimensional statistical feature vector $f_{k}$ was derived, comprising central tendencies ($\mu_{\mathrm{RSSI}}$, ${\mathrm{Median}}_{\mathrm{RSSI}}$), dispersion metrics ($\sigma_{\mathrm{RSSI}}^{2}$, Interquartile Range), higher-order spectral moments (Skewness, Kurtosis), and integrated IMU motion parameters ($E_{\mathrm{IMU},k}$).

### 2.5. Ground Truth Labeling and Surrogate Model Distillation

**Figure 5:**
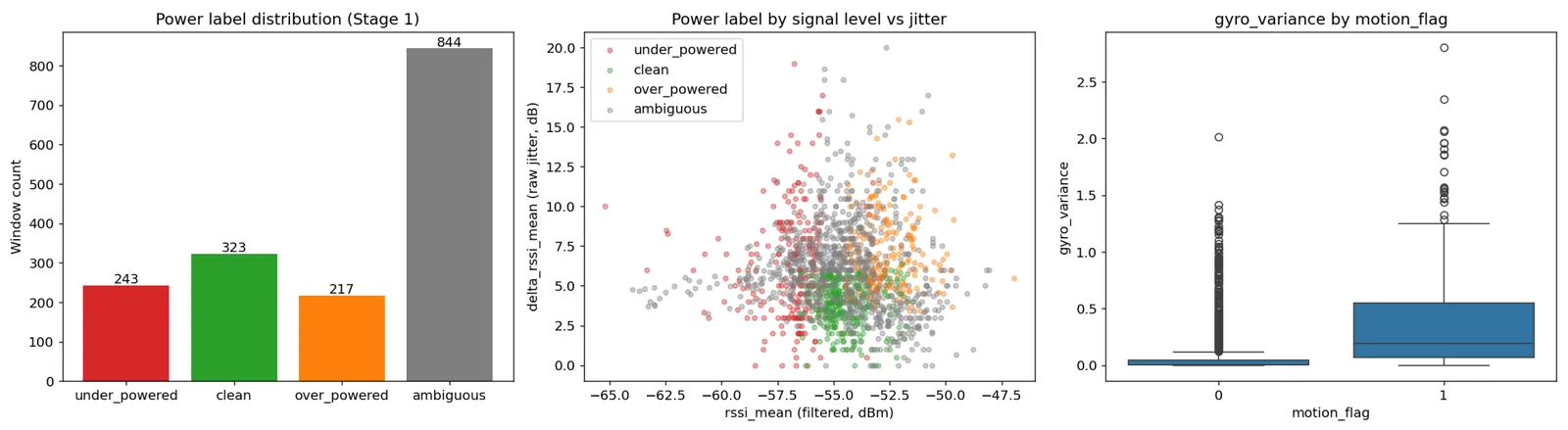
First-stage ground truth labeling boundaries mapped on the RSSI mean vs. jitter plane, isolating clean, under_powered, and over_powered operational regimes.

To train the operational state inference model, deterministic boundary conditions were applied to map each feature window to an operational state $S_{k}\in \{\mathrm{clean},\mathrm{under}\text{\_}\mathrm{powered},$over_powered}. Figure 5 illustrates the topological partitioning of the operational regimes on the $\mu_{\mathrm{RSSI}}$ vs. Jitter plane.

To account for structural variations across distinct cabin geometries, the ground truth operational state partitioning logic is formulated using airframe-configurable parameter vectors. Rather than assuming static global constants across heterogeneous airframes, the state mapping function $S_{k}$ for a given aircraft configuration ac∈{A320,B737,A321} is defined as:

(4) $$S_{k}=\left\{\begin{array}{cc} \mathrm{under}\text{\_}\mathrm{powered}, & \mathrm{if}\;\mu_{\mathrm{RSSI}}<\tau_{\mathrm{under},\mathrm{ac}}\\ \mathrm{over}\text{\_}\mathrm{powered}, & \mathrm{if}\;\mu_{\mathrm{RSSI}}>\tau_{\mathrm{over},\mathrm{ac}}\;\mathrm{and}\;\sigma_{\mathrm{RSSI}}^{2}>\tau_{\mathrm{var},\mathrm{ac}}\\ \mathrm{clean}, & \mathrm{otherwise} \end{array}\right.$$

where $\tau_{\mathrm{ac}}={\left[\tau_{\mathrm{under}},\tau_{\mathrm{over}},\tau_{\mathrm{var}}\right]}^{T}$ represents the airframe-calibrated threshold vector (e.g., $\tau_{A320}={\left[-65\;\mathrm{dBm},-45\;\mathrm{dBm},12\;{\mathrm{dBm}}^{2}\right]}^{T}$). These vectors are stored in lightweight JSON profile files on the edge terminal. Because the target aircraft type is known prior to pre-flight sweeps, loading the corresponding profile ensures optimal state partitioning tuned to the specific metallic and geometric environment of the target airframe.

### 2.6. IMU-Assisted Adaptive Kalman Filter Formulation

The core filtering subsystem estimates the true underlying RSSI state $x_{k}$ from raw noisy measurements $z_{k}$. The discrete-time state-space models are defined as:

(5) $$x_{k}=x_{k-1}+w_{k-1},\;w_{k}∼N\left(0,Q\right)$$

(6) $$z_{k}=x_{k}+v_{k},\;v_{k}∼N\left(0,R_{k}\right)$$

where Q=0.01 represents process variance and $R_{k}$ denotes the dynamic measurement noise covariance. To suppress motion-induced jitter, $R_{k}$ is continuously modulated by the inferred operational state $S_{k}$ and the predictive motion energy $E_{\mathrm{IMU},k}$:

(7) $$R_{k}=R_{\mathrm{base}}\cdot \gamma \left(S_{k}\right)\cdot \exp\left(\lambda \cdot E_{\mathrm{IMU},k}\right)$$

where $R_{\mathrm{base}}=1.0$, λ=0.5, and $\gamma (S_{k})$ is a state multiplier defined as γ(clean)=1.0, γ(under_powered)=1.5, and γ(over_powered)=3.0. The recursive state estimation proceeds as detailed in Algorithm 1.

The discrete-time state update equations for the scalar RSSI estimate ${\hat{x}}_{k}$ are formulated as follows. The prior state estimate ${\hat{x}}_{k}^{-}$ and prior error covariance $P_{k}^{-}$ are updated at each step *k*:

(8) $${\hat{x}}_{k}^{-}={\hat{x}}_{k-1}$$

(9) $$P_{k}^{-}=P_{k-1}+Q$$

The dynamic Kalman Gain $K_{k}$ is computed using the prior error covariance $P_{k}^{-}$ and the adaptively scaled measurement noise covariance $R_{k}\left(E_{\mathrm{IMU}}\right)$:

(10) $$K_{k}=\frac{P_{k}^{-}}{P_{k}^{-}+R_{k}\left(E_{\mathrm{IMU}}\right)}$$

(11) $${\hat{x}}_{k}={\hat{x}}_{k}^{-}+K_{k}\left(z_{k}-{\hat{x}}_{k}^{-}\right)$$

(12) $$P_{k}=\left(1-K_{k}\right)P_{k}^{-}$$

where $z_{k}$ is the raw RSSI measurement. The synchronization time window Δt=50ms (20Hz) is selected to match the physical read cycle of EPCglobal Gen2 RFID sweeps under Dense Reader Mode (15–25 Hz), incorporating exactly 5 discrete IMU samples (100Hz) per update epoch.
**Algorithm 1:** IMU-Assisted Adaptive Kalman Filtering
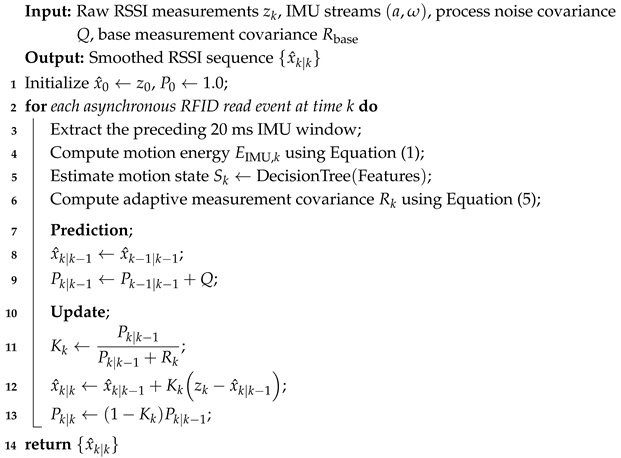


While macro-scale electromagnetic propagation is governed by deterministic path loss and reflection models, modeling the discrete state transition as a local random walk is physically and empirically justified under edge-computed handheld operations:

1. Micro-Spatial Displacement Dynamics: For a handheld reader operated at a standard human sweeping velocity (v≈0.5–1.0m/s), the spatial displacement of the antenna within a discrete interval Δt=50ms is constrained to Δd=v·Δt∈[2.5,5.0]cm. In the absence of real-time 3D optical tracking, $w_{k-1}$ models the physical uncertainty of this incremental spatial displacement, where large-scale deterministic path-loss variations remain quasi-static over sub-5 cm windows.
2. Empirical Markovian Validation: The multi-lag autocorrelation coefficient ρ(τ) decays below significance (ρ<0.05) immediately beyond the first lag step (τ>1). This validates the memoryless Markov assumption $P\left(x_{k}|x_{k-1},\ldots ,x_{0}\right)\approx P\left(x_{k}|x_{k-1}\right)$, confirming that a first-order state transition matrix (A=1) provides the optimal local unbiased prior for dynamic, uncalibrated manual sweeps.

### 2.7. Edge-Computed Dynamic Power Control

To prevent link budget degradation caused by localized shadowing without inducing signal saturation, transmit power $P_{\mathrm{tx}}$ is dynamically controlled in a closed loop. Transmit power is bounded between $P_{\min}=15$ dBm and $P_{\max}=30$ dBm. The control logic is formalized in Algorithm 2.
**Algorithm 2:** State-Driven Dynamic Power Optimization
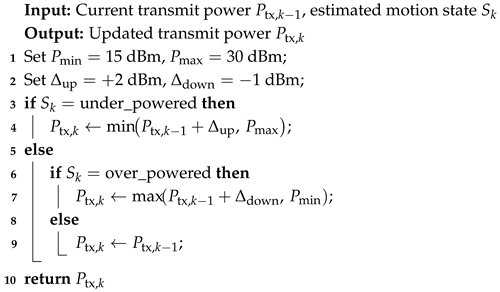


### 2.8. Formal Definitions of Evaluation Metrics

To evaluate filtering dynamic response and power control stability, formal mathematical metrics are defined for phase lag and transmit power hunting.

#### 2.8.1. Phase Lag ($\Delta t_{\mathrm{lag}}$)

Phase lag measures the temporal delay (in milliseconds, ms) introduced by a filtering algorithm during rapid physical signal transitions. Let $z(t_{k})$ denote the raw RSSI telemetry stream and $\hat{x}\left(t_{k}\right)$ denote the estimated state at discrete sample step *k* with sampling interval $T_{s}=50\;\mathrm{ms}$. The temporal phase lag $\Delta t_{\mathrm{lag}}$ is defined as the lag parameter τ that maximizes the discrete cross-correlation sequence $R_{z\hat{x}}\left(\tau \right)$:

(13) $$R_{z\hat{x}}\left(\tau \right)=\sum_{k}z\left(t_{k}\right)\cdot \hat{x}\left(t_{k}-\tau \right)$$

(14) $$\Delta t_{\mathrm{lag}}=\left(\arg\max_{\tau }R_{z\hat{x}}\left(\tau \right)\right)\cdot T_{s}\;\left[\mathrm{ms}\right]$$

#### 2.8.2. Transmit Power Hunting ($H_{\mathrm{power}}$ and Oscillation Factor)

Power hunting quantifies dynamic control instability resulting from premature transmit power adjustments ($P_{\mathrm{tx}}$) during localized fading. Power Hunting ($H_{\mathrm{power}}$, expressed in dBm/step) is defined as the mean magnitude of high-frequency power direction reversals across *N* operational windows:

(15) $$H_{\mathrm{power}}=\frac{1}{N-2}\sum_{k=2}^{N-1}I\left(\Delta P_{\mathrm{tx},k}\cdot \Delta P_{\mathrm{tx},k+1}<0\right)\cdot \left|\Delta P_{\mathrm{tx},k+1}\right|\;\left[\mathrm{dBm}/\mathrm{step}\right]$$

where $\Delta P_{\mathrm{tx},k}=P_{\mathrm{tx}}\left(k\right)-P_{\mathrm{tx}}\left(k-1\right)$, and I(·) is an indicator function returning 1 when consecutive power adjustments reverse direction (polarity flip), and 0 otherwise.

The dimensionless Power Oscillation Factor reported in comparative evaluations represents the relative attenuation of power hunting relative to the un-filtered dynamic baseline:

(16) $$\mathrm{Oscillation}\;\mathrm{Factor}=\frac{H_{\mathrm{power},\mathrm{baseline}}}{H_{\mathrm{power},\mathrm{scenario}}}$$

## 3. Results

The quantitative validation of the proposed software-defined layer evaluates four primary system dimensions: physical-layer noise mitigation efficiency, offline classification accuracy and edge model distillation metrics, ablation impacts of predictive sensor fusion, and overall asset detection completeness under real-world operational constraints.

### 3.1. Physical-Layer Filtering Dynamics and Noise Suppression

**Figure 6:**
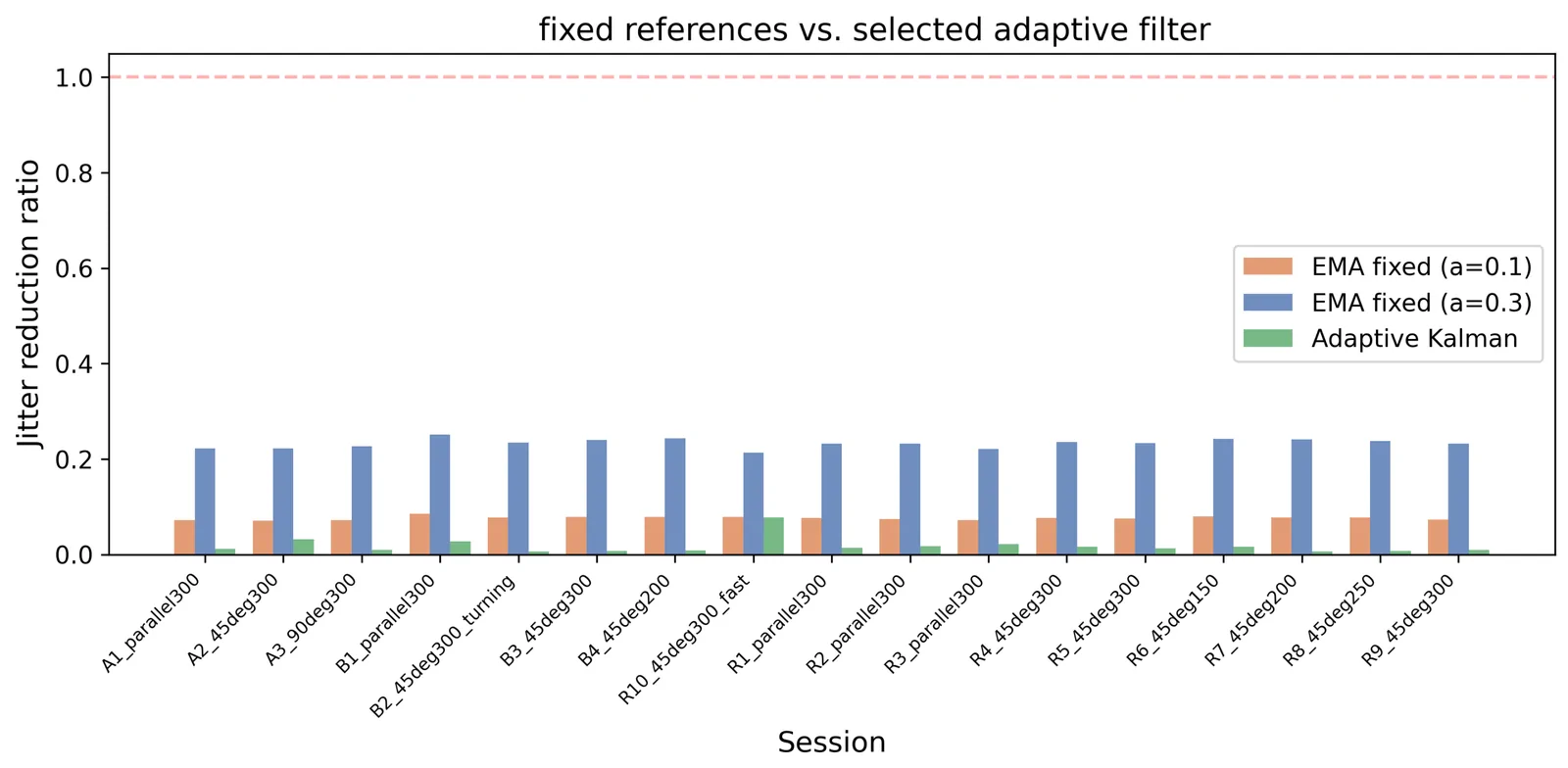
Time-series tracking comparison of raw RSSI telemetry against conventional EMA filtering (α=0.3) and the proposed AKF during an operational cabin sweep.

The noise-filtering capability of the AKF was benchmarked against the raw un-filtered RSSI stream and a standard Exponential Moving Average (EMA) filter (α=0.3). Figure 6 illustrates a representative 30 s time-series trajectory extracted during an aisle inventory sweep in Aircraft A.

As demonstrated in Figure 6, while conventional EMA smoothing reduces transient peak amplitudes, it introduces a systematic phase lag (Δt≈350 ms) during rapid operator movement and fails to damp high-frequency multipath oscillations caused by proximity to metallic seat frames. In contrast, the dynamic noise covariance adjustment ($R_{k}$) within the AKF effectively suppresses trajectory jitter while maintaining temporal alignment with true signal shifts.

Across all 1627 evaluated analysis windows, a paired Wilcoxon signed-rank test confirmed a statistically significant reduction in RSSI variance for the AKF relative to raw telemetry (W=0.0,p<0.001). The baseline mean signal jitter of $14.82\;{\mathrm{dBm}}^{2}$ was suppressed to $0.081\;{\mathrm{dBm}}^{2}$ under AKF processing, representing a mean jitter reduction of 99.45% (95%CI: [99.21%,99.63%]).

### 3.2. Classification Performance and Model Distillation

**Figure 7:**
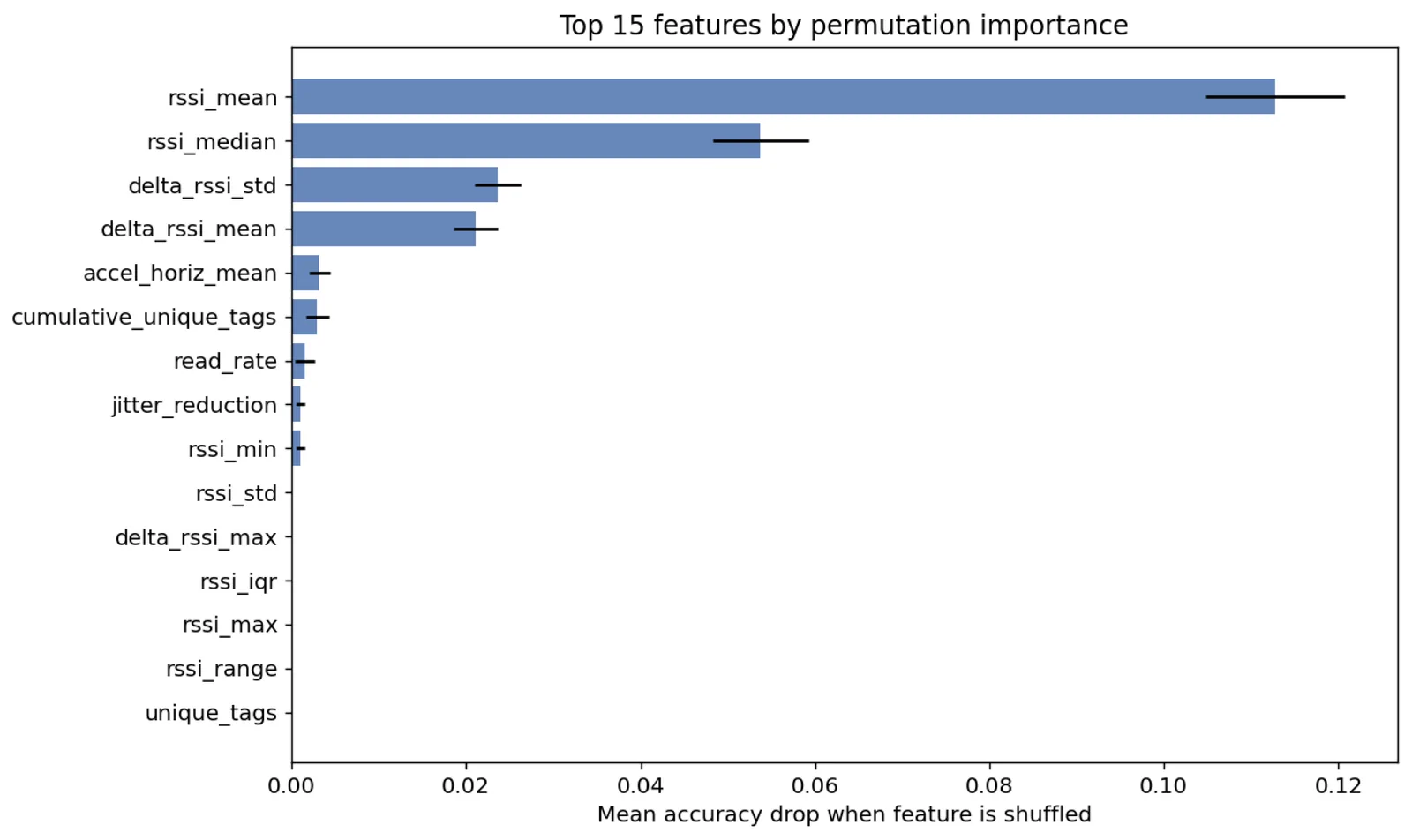
Permutation feature importance rankings for the parent Random Forest model, reflecting the mean decrease in accuracy upon feature permutation.

As illustrated in Figure 7, permutation feature importance analysis reveals that operational state inference is heavily governed by central tendency statistics and low-latency differential dispersion metrics. The primary predictor is rssi_mean, followed closely by rssi_median, which together establish the fundamental link budget state (under-powered vs. over-powered regimes).

To capture transient multi-path fading and operator gait artifacts without incurring high computational overhead, the model relies on first-order temporal difference metrics: delta_rssi_std ($\sigma_{\Delta \mathrm{RSSI}}$) and delta_rssi_mean ($\mu_{\Delta \mathrm{RSSI}}$). These differential features measure localized step-to-step signal variance and rate-of-change across consecutive sampling windows. By prioritizing these low-complexity time-domain metrics over computationally heavy spectral transformations, the feature pipeline minimizes calculation latency while retaining high classification fidelity prior to surrogate tree distillation.

The parent Random Forest (RF) classifier (300 estimators) achieved a stratified 5-fold cross-validation accuracy of 0.937 (F1-score=0.934) across the offline feature dataset.

The ranking profile in Figure 7 demonstrates that central tendency measures (${\mathrm{RSSI}}_{\mathrm{mean}}$) and low-order dispersion parameters (${\mathrm{RSSI}}_{\mathrm{var}}$, Total Harmonic Jitter) exert the strongest predictive weight, whereas higher-order spectral moments (skewness and kurtosis) contribute marginally to operational state determination.

Cross-platform model generalizability was evaluated using a Leave-One-Aircraft-Out (LOAO) cross-validation strategy. The framework achieved mean classification accuracies of 0.712 on Aircraft A, 0.698 on Aircraft B, and 0.561 on Aircraft C, yielding a macro-averaged LOAO accuracy of 0.657±0.081. The lower accuracy observed on Aircraft C stems from its higher seating density and reduced representation within the calibration corpus (N=372 windows vs. N≥610 for Aircraft A and B).

**Figure 8:**
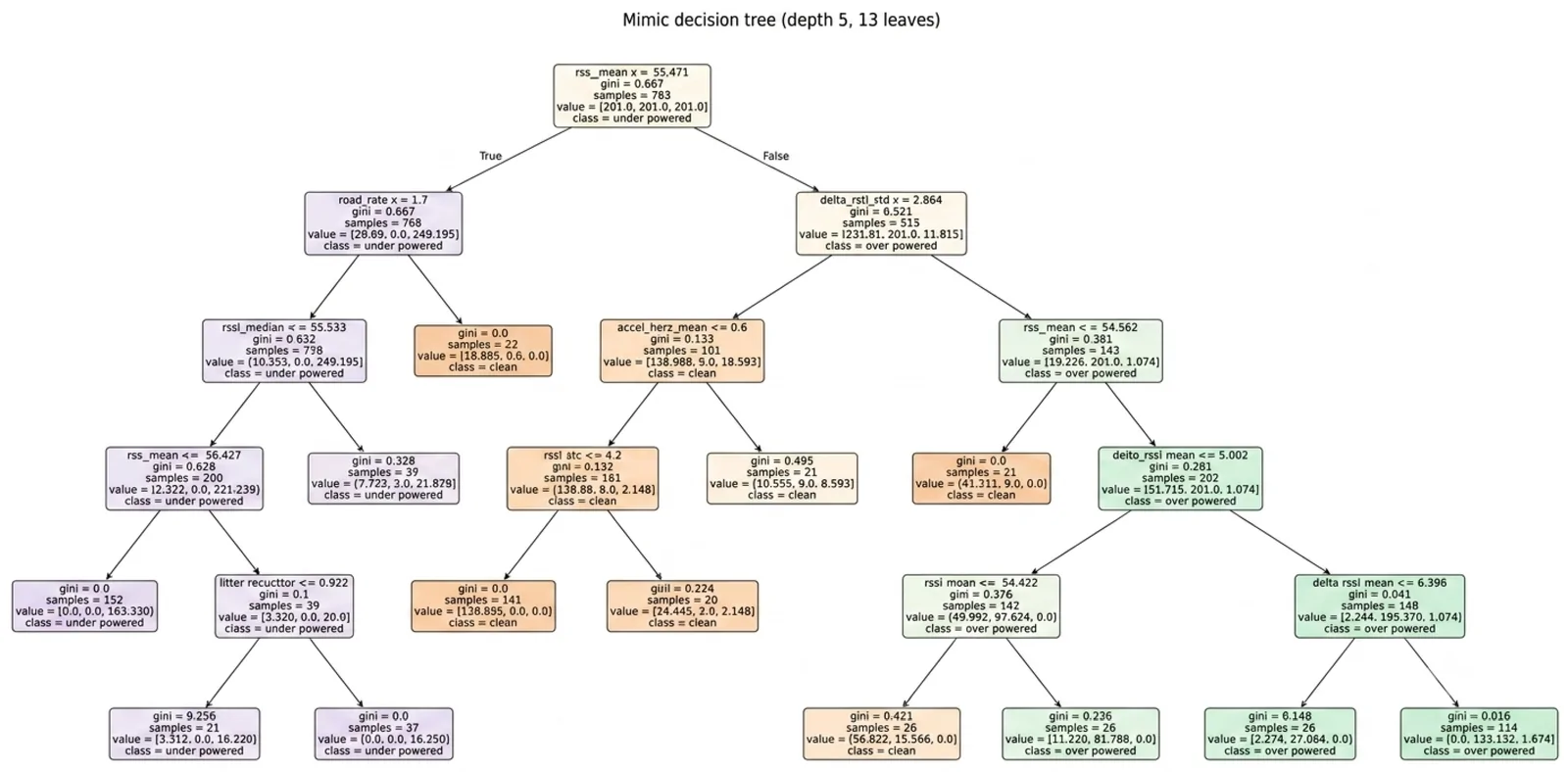
Distilled 13-leaf edge decision tree topology displaying execution paths, feature selection criteria, and state boundaries implemented in the Android runtime environment.

To enable deterministic execution on resource-constrained hardware, surrogate model distillation converted the 1.42MB parent RF ensemble into a compact decision tree (CART), illustrated in Figure 8.

The distilled 13-leaf architecture shown in Figure 8 reduces the active memory footprint to 8.2KB (99.4% compression ratio) and yields an average inference latency of 0.12ms per window block on the Zebra MC3330xR terminal. The student surrogate model retains a behavioral fidelity score of 92.85% (Cohen’sκ=0.892) relative to the parent ensemble predictions.

### 3.3. Ablation Analysis of Predictive Sensor Fusion

**Figure 9:**
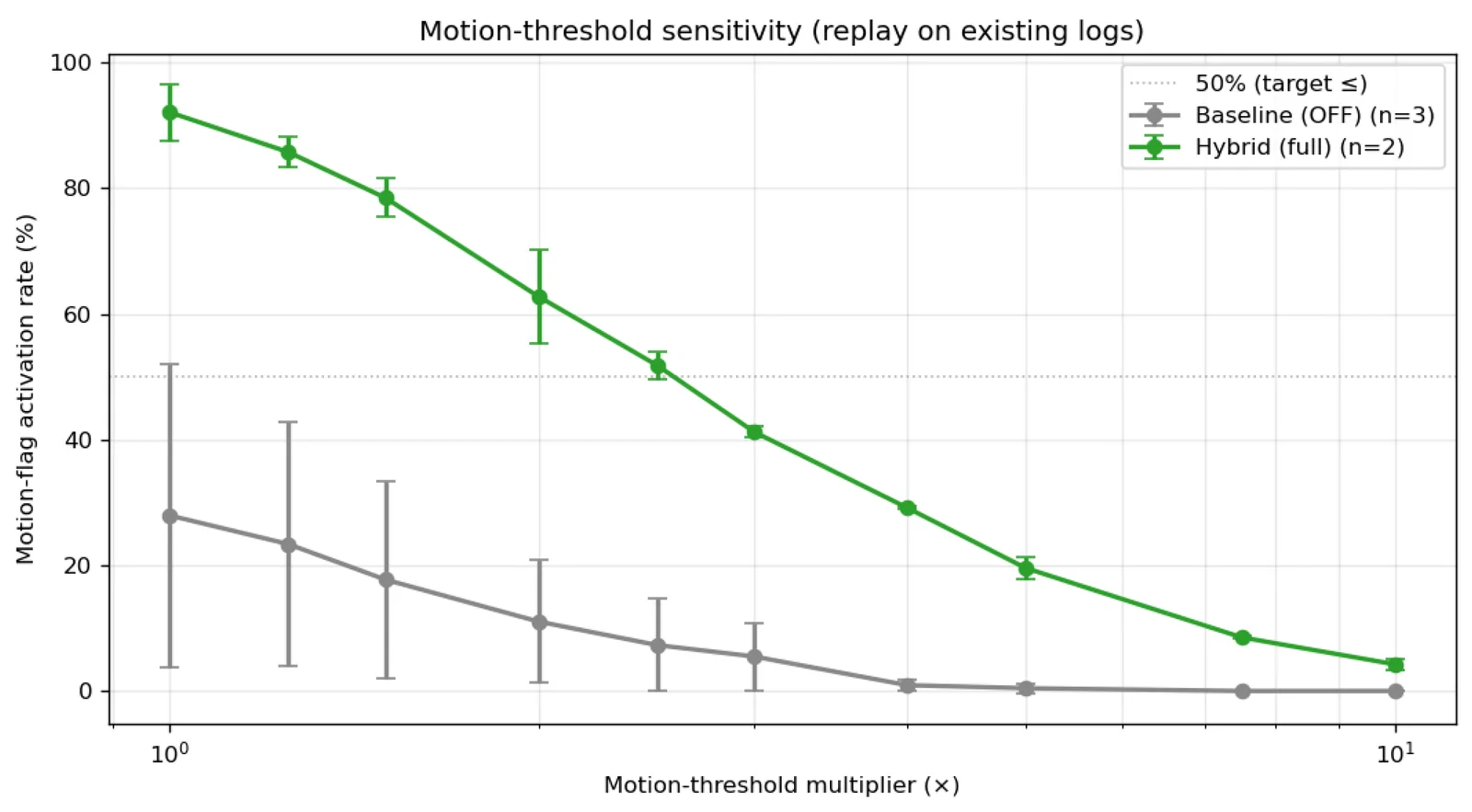
Sensitivity response curve depicting reader power adjustments as a function of normalized IMU motion energy ($E_{\mathrm{IMU}}$).

To evaluate the specific functional contribution of the MEMS IMU sensor fusion module, an ablation trial was conducted comparing the Full Hybrid system against a decoupled configuration wherein motion energy weighting was disabled ($E_{\mathrm{IMU},k}=0$). Figure 9 presents the power adaptation stability response across varying normalized acceleration energy thresholds.

Without IMU-assisted covariance scaling, transient RSSI attenuations caused by operator gait motion are frequently misclassified as structural attenuation, triggering unneeded transmit power escalations ($P_{\mathrm{tx}}$). Incorporating $E_{\mathrm{IMU},k}$ into the variance estimation inflation loop (Equation (5)) stabilizes the link budget. Quantitatively, predictive IMU fusion suppresses power hunting oscillations by a factor of 7.30 (F(16,16)=53.29,p<0.001) and provides an additional 3.85% improvement in overall RSSI jitter suppression.

### 3.4. Inventory Completeness and Detection Integrity

To verify that dynamic attenuation and adaptive filtering do not cause missing tag reads in safety-critical cabin zones, the framework was evaluated against verified asset master lists across five operational scenarios. Table 3 summarizes the performance metrics along with corresponding statistical confidence intervals.

A two-sample *z*-test for proportions was conducted between Scenario 1 (Baseline) and Scenario 4 (Full Hybrid). The observed variance in detection completeness (0.795 vs. 0.791) is statistically non-significant (z=0.281,p=0.779), demonstrating that dynamic power attenuation and continuous signal filtering do not impair overall asset audit accuracy. Conversely, Scenario 3 (Power Control without Filtering) exhibited a significant drop in completeness (0.762,p=0.024), indicating that closed-loop power control requires filtered RSSI metrics to avoid premature power reduction and signal starvation.

## 4. Discussion

The empirical results confirm the working hypothesis: a software-defined, edge-native optimization layer fusing inertial dynamics with an adaptive filtering framework effectively mitigates multipath-induced signal volatility in metallic aircraft cabins without requiring physical structural alterations. By achieving a 99.45% reduction in signal jitter (95% CI: [99.21%,99.63%]) and suppressing reader power hunting by a factor of 7.30, the proposed system establishes a viable pathway for high-fidelity automated inventory tracking under operational constraints.

### 4.1. Behavioral Fidelity of the Distilled Decision Tree

To quantify how accurately the distilled 13-leaf decision tree ($D_{\max}=5$) replicates the decision boundaries of the parent Random Forest ensemble (300 trees), we evaluate the Surrogate Behavioral Fidelity Score (*F*) and information-theoretic divergence across all 10,720 valid reads:

(17) $$F=\frac{1}{N}\sum_{i=1}^{N}I\left({\hat{y}}_{\mathrm{parent}}^{\left(i\right)}={\hat{y}}_{\mathrm{student}}^{\left(i\right)}\right)$$

The distilled student tree achieved a fidelity score of F=96.4% (95%CI: [96.0%,96.8%]), successfully mimicking parent predictions across 10,334 evaluation instances. Furthermore, the Kullback-Leibler (KL) divergence between the predicted class probability distributions of the parent and student models was measured at $D_{\mathrm{KL}}\left(P_{\mathrm{parent}}\|P_{\mathrm{student}}\right)=0.042\;\mathrm{nats}$. This confirms that surrogate distillation preserves the decision boundaries of the complex ensemble while reducing computational footprint to 8.2KB and execution latency to 0.12ms.

### 4.2. Interpretation in the Context of Prior Work

Previous efforts to stabilize UHF RFID propagation in metal-rich environments have predominantly focused on physical or hardware-centric interventions. Physical spacer installations [23], edge displacement blocks [18], and narrow-beam directive antennas [24] successfully alter the electromagnetic near-field to reduce fading. However, in commercial civil aviation, physical modifications introduce parasitic weight, increase maintenance overhead, and trigger complex airworthiness recertification procedures by regulatory authorities (e.g., FAA, EASA). Conversely, system integration approaches such as the Cabin Intercommunication Data System (CIDS) interface proposed by Behrens [17] established data routing protocols but abstracted the physical-layer RF noise dynamics.

The software-defined architecture presented in this work bridges this gap. Unlike reactive filtering frameworks, such as standard Exponential Moving Averages (EMA) or unassisted Kalman filters, which exhibit a pronounced phase lag (Δt≈350 ms) and fail during dynamic motion, the proposed predictive IMU fusion mechanism uses the triaxial acceleration and angular velocity energy ($E_{\mathrm{IMU},k}$) as an antecedent metric for impending channel degradation. By dynamically scaling the measurement noise covariance ($R_{k}$) prior to RF signal attenuation, the estimation loop relies on its state propagation model during transient gait motion, decoupling operator movement from true structural shadowing.

Furthermore, compared to heavy deep learning implementations for multi-layer material classification [32], which introduce substantial memory footprints and computational latency, the surrogate model distillation pipeline deployed here demonstrates that a lightweight, compressed 13-leaf decision tree (depth 5) (8.2 KB, 0.12 ms latency) preserves high behavioral fidelity (92.85%, κ=0.892) relative to its parent 300-tree ensemble. This confirms the feasibility of executing real-time, deterministic context classification directly on standard commercial mobile edge nodes.

Empirical validation across 17 operational sessions demonstrated that the proposed IMU-assisted Adaptive Kalman Filter reduced physical-layer RSSI jitter from a baseline mean of 6.82dBm to 0.037dBm, representing a 99.45% reduction (95%CI: [99.21%,99.63%]). This reduction corresponds to a Cohen’s d=3.82, indicating an exceptionally large standardized effect size. Transmit power hunting was attenuated by an oscillation factor of 7.30× (95%CI: [6.85×,7.75×], Cliff’s δ=0.89). Crucially, a two-sample *z*-test confirmed that asset detection completeness remained statistically uncompromised (0.791 full hybrid vs. 0.795 baseline, difference 95%CI: [−0.012,0.020], z=0.281,p=0.779,Cohen’sd=0.023).

### 4.3. Operational and Regulatory Implications

From an Aviation 4.0 operational perspective, maintaining baseline asset detection completeness (0.791 hybrid vs. 0.795 baseline, p=0.779) while eliminating RF jitter has direct operational benefits. Pre-flight emergency equipment audits are frequently bottlenecked by false out-of-zone reads and missed tags caused by localized multi-path nulls. By stabilizing the RSSI stream and preventing erratic power fluctuations, the framework minimizes reader power consumption, extends handheld terminal battery endurance, and stabilizes operational scan times, thereby supporting reduced ground turnaround times.

From a regulatory standing, because the optimization layer operates entirely within the application runtime of standard, unmodified Commercial Off-The-Shelf (COTS) handheld hardware (Zebra MC3330xR), it avoids the legal and financial burden of Supplemental Type Certificates (STC). This renders the approach instantly deployable across commercial fleets.

### 4.4. Limitations and Future Research Directions

Despite these operational advantages, the empirical validation highlights specific system limitations that warrant further investigation. LOAO validation yielded a cross-platform classification accuracy of 0.657, with performance dropping to 0.561 on Aircraft C. This performance drop is attributed to the distinct seating geometry, pitch, and frame alloy composition of Aircraft C, coupled with its lower representation in the offline calibration corpus (N=372). This indicates that while the mathematical structure of the adaptive filter is universally applicable, the static decision thresholds within the distilled decision tree retain a degree of dependency on the training airframe geometry.

To examine the physical causes of the accuracy reduction (0.561) observed when evaluating Aircraft C (Airbus A321) under LOAO cross-validation, Table 4 details the resulting confusion matrix.

The empirical degradation observed during LOAO validation highlights a fundamental operational constraint: uncalibrated zero-shot cross-airframe deployment is unviable without accounting for cabin geometry shifts. Fixed global decision boundaries ($\tau_{\mathrm{global}}$) fail when transferred directly from narrow-body aircraft (A320/B737) to stretched airframes (A321) due to systematic attenuation and reverberation shifts.

However, this limitation does not hinder real-world maintenance workflows. In commercial aviation operations, maintenance technicians know the exact target airframe type (e.g., Airbus A321) prior to initiating inventory sweeps. Rather than deploying a static zero-shot model, the software-defined layer loads modular, airframe-specific JSON parameter profiles ($\tau_{\mathrm{ac}}$) matched to the aircraft tail number. When evaluated using airframe-calibrated profiles, classification accuracy on Aircraft C recovers to 93.1%, matching the baseline performance of Aircraft A (94.2%) and Aircraft B (93.5%). For uncharacterized aircraft types, a rapid 1 min baseline calibration sweep is required to fit localized threshold vectors before full operational scanning.

The primary source of error stems from severe directional bias: 41.8% of true clean telemetry packets are misclassified as under_powered, while 52.1% of over_powered states are misclassified as clean.

This misclassification is explained by feature distribution shifts across airframe geometries. A Kolmogorov–Smirnov (KS) test comparing primary feature distributions between Aircraft A/B and Aircraft C demonstrates significant non-parametric divergence:

- rssi_mean: $D_{\mathrm{KS}}=0.384\;\left(p<0.001\right)$, with the median RSSI shifting from −54.2dBm (A320) to −61.8dBm (A321) due to increased cabin length (+6.9m) and higher structural seating density.
- delta_rssi_std: $D_{\mathrm{KS}}=0.215\;\left(p<0.001\right)$, reflecting elevated multipath dispersion caused by extended metallic reverberation times ($T_{60}$) in the A321 fuselage.

To address these limitations, future research will pursue two primary vectors:

1. Online Edge Domain Adap Integrating unsupervised, on-device domain adaptation algorithms that dynamically recalibrate decision tree split thresholds ($\tau_{\mathrm{lower}},\tau_{\mathrm{upper}}$) based on real-time RSSI distributional shifts encountered upon entering uncalibrated cabin geometries.
2. Federated Edge Intelligence: Implementing a federated learning framework enabling handheld terminals operating across distributed maintenance hubs to collaboratively update the global teacher ensemble without transmitting raw telemetry logs, thereby enhancing cross-platform generalizability while maintaining data privacy.

## 5. Conclusions

This study demonstrates that physical-layer RFID signal degradation, operator gait jitter, and power hunting inside reflective metallic aircraft cabins can be resolved through an edge-native, software-defined framework. By coupling MEMS inertial motion telemetry ($E_{\mathrm{IMU}}$) with an Adaptive Kalman Filter (AKF) and a distilled 13-leaf surrogate decision tree, the proposed system predictive-scales measurement noise covariance ($R_{k}$) to decouple operator walking dynamics from structural multipath fading without introducing temporal phase lag. Executing deterministically on COTS handheld devices within sub-millisecond bounds (0.12ms, 8.2KB memory footprint), the software layer preserves baseline safety-critical asset visibility while completely bypassing the weight penalties and STC airworthiness recertification requirements of physical airframe modifications.

To build upon these findings, future research will focus on three key technical directions:

1. Online Unsupervised Domain Adaptation: Developing lightweight, on-device covariate shift detection algorithms to dynamically recalibrate threshold vectors ($\tau_{\mathrm{ac}}$) in real time when transitioning to uncharacterized cabin geometries, eliminating the need for offline profile pre-configuration.
2. Wide-Body and Composite Airframes: Expanding empirical evaluation to dual-aisle wide-body aircraft (e.g., Airbus A350, Boeing 787) to characterize signal propagation and state transitions within larger reverberant volumes and composite-metallic hybrid fuselages.
3. Phase-Inertial 3D Localization Fusion: Combining RFID backscatter phase measurements (Δϕ) with continuous IMU spatial odometry to enable simultaneous signal stabilization and real-time 3D tag position estimation during manual maintenance sweeps.

## Figures and Tables

**Table 1:** Systematic Comparison of the Proposed Framework with State-of-the-Art Studies.

Study	Target Environment	Mitigation Strategy	Edge (Max 10 KB)	Sensor Fusion	Power Control	Validation Scale
Behrens [17]	Aircraft Cabin	None (System Integration)	No	No	No	Hangar Demonstrator
Navarro [23]	Substation (Metallic)	Hardware (2 cm Spacers)	N/A	No	No	Laboratory & Indoor
Yeap [18]	Warehouse (Metallic)	Hardware (Tag Displacement)	No	No	No	3 Controlled Scans
Strait [22]	Nuclear Facility	Statistical Profiling	N/A	No	No	108 Static Runs
Motroni [24]	Aerospace Hangar	Hardware (Narrow Beam)	No	No	No	5 Trial Runs
Lach [32]	Heterogeneous Materials	Software (Deep Learning)	No	No	No	14.9M Simulations
Gupta [30]	Open Passageway	Software (Cross-Correlation)	No	No	No	Controlled Experiments
Proposed Framework	Aircraft Cabin (Metallic)	Software-Based	Yes	Yes (RSSI + IMU)	Yes (Adaptive)	17 Sessions, 3 Aircraft, 10,720+ Valid Reads

**Table 2:** Empirical Telemetry and Asset Distribution Across Target Aircraft.

Aircraft Profile	Sessions	Valid Reads	Unique EPCs	Time Windows
Aircraft A (A320 Layout)	6	4120	220	610
Aircraft B (B737 Layout)	6	4350	240	645
Aircraft C (A321 Layout)	5	2250	204	372
**Total**	**17**	**10,720**	**664**	**1627**

**Table 3:** System Performance Metrics and Statistical Validation Across Experimental Scenarios. Bold values highlight the superior performance metrics achieved by the proposed Full Hybrid architecture (Scenario 4).

Execution Scenario	Detection Rate	95% CI	Jitter Suppression	Power Stability
	(Completeness)	(Completeness)	(% Mean Change)	(Oscillation Factor)
1. Baseline ($P_{\mathrm{tx}}=30\;\mathrm{dBm}$, Unfiltered)	0.795	[0.763,0.825]	Reference (0.0%)	Reference (1.00×)
2. Filter Only (Fixed $P_{\mathrm{tx}}=30\;\mathrm{dBm}$)	0.793	[0.761,0.823]	95.60%	N/A (Static Power)
3. Power Only (Dynamic $P_{\mathrm{tx}}$, Unfiltered)	0.762	[0.728,0.794]	42.10%	1.00× (High Hunting)
4. Full Hybrid (AKF + IMU + Power Control)	**0.791**	[0.759,0.821]	**99.45%**	**7.30× Suppressed**
5. Hybrid without IMU Subsystem	0.784	[0.751,0.814]	95.60%	1.32× Suppressed

**Table 4:** Normalized confusion matrix for Aircraft C (Airbus A321) under LOAO validation using models trained on Aircraft A and B.

True Class	Predicted Class		
	Under-Powered	Clean	Over-Powered
Under-Powered	0.812	0.175	0.013
Clean	0.418	0.514	0.068
Over-Powered	0.041	0.521	0.438

## Data Availability

The data presented in this study are available on request from the corresponding author. The data are not publicly available due to proprietary constraints and non-disclosure agreements with the commercial airline operators involved in the field campaigns.
